# Breeding for sheep robustness: simulation of the consequences of ewe-lamb energy allocation trade-offs

**DOI:** 10.1186/s12711-026-01047-8

**Published:** 2026-04-26

**Authors:** Maya Hiltpold, Ronan Trépos, Frédéric Douhard

**Affiliations:** 1 GenPhySE, Université de Toulouse, INRAE, ENVT, 31326 Castanet-Tolosan, France; 2 Present Address: ProSpecieRara, Hellgasse 1, 5103 Wildegg, Switzerland; 3 INRAE, Université de Toulouse, UR MIAT, Auzeville-Tolosane, France

## Abstract

**Background:**

Sheep farmed in grazing systems are usually exposed to seasonal feed quality fluctuations that are likely to increase with climate change. In this context, a major challenge is to breed robust ewes, able to sustain a high lifetime performance in a large range of feeding conditions. However, trade-offs can exist between components of lifetime performance, in particular due to energy allocation constraints at the individual level. Here we focus on predicting ewe-lamb trade-offs that correspond to the competitive energy allocation between the mother and her lambs until weaning. We explored the consequences of those trade-offs on lifetime performance (i.e. the total number of lambs weaned) using a bioenergetic model coupling the ewe and her lambs. To this end, we ran a global sensitivity analysis of our model to a set of input parameters differentiating “individual types” in terms of energy allocation mechanisms, and in two contrasted grassland-based feed environment scenarios.

**Results:**

The unfavorable scenario, which included poor grass and forage quality due to summer drought, resulted in an average reduction of about 37% in the total number of lambs weaned compared with the favorable, high feed quality scenario. This lifetime performance was only moderately correlated between the two environmental scenarios. A re-ranking of individual types occurred across environments and was driven by a re-ranking in neonatal survival and ewe longevity whereas ewe prolificacy showed little environmental sensitivity. The ewe-lamb trade-offs assumed at the individual level did not lead to the negative consequences expected among-individuals: at this level, the correlations between ewe longevity and prolificacy or lamb survival were close to zero in both environments.

**Conclusions:**

Provided that energy allocation trade-offs are under genetic control, they could underpin a genotype-by-environment interaction in ewe lifetime performance. However, energy allocation constraints alone do not exclude the existence of robust ewes that manage well the consequences of ewe-lamb trade-offs, in particular by combining high target levels of intake and body condition.

**Supplementary Information:**

The online version contains supplementary material available at 10.1186/s12711-026-01047-8.

## Introduction

Selection for high animal productivity under conditions favoring the full expression of the genetic potential [1] has driven tremendous gains in various livestock productions but probably also eroded animal robustness [2]. Consequently, the physiological capacities of animals selected under favorable conditions may not fit well with farm environments that provide constraining conditions. In extensive pasture and grassland-based systems, robust animals that are able to cope with constrained and variable feed will be increasingly needed because of increased frequency of drought and extreme weather conditions with climate change [3]. To support breeding strategies on robustness, the assessment of genotype-by-environment (G × E) interactions on a large range of production environments is then advocated [4].

Yet, the magnitude of G × E interactions is variable among traits and the definition of traits that could adequately reflect animal robustness remains an issue. Therefore, a systemic definition of robustness that integrates multiple traits reflecting the animal ability to overcome environmental challenges faced throughout their entire life is of particular interest [5]. A key argument for considering lifetime performance is the evolutionary assumption that trade-offs exist between robustness components, in particular between current reproduction and future reproduction or survival [6]. When those life functions compete for the use of nutrients, an organism is assumed to face resource allocation trade-offs, a situation which in livestock science is usually viewed from the physiological perspective of nutrient partitioning mechanisms [7]. At present, it remains uncertain whether trade-offs between reproduction and survival are ubiquitous, and whether robust individuals can be genetically characterized by their energy allocation [8, 9].

In suckler ewes, energy allocation to current reproduction (i.e. pregnancy and lactation) is largely determined by ewe prolificacy. Litter size is of economic interest in general [10]. However, the most prolific ewes can be at risk of a reduced longevity, poor lamb survival, and are thus not necessarily those with the highest lifetime performance (e.g. total number of lambs weaned over the ewe life) [11, 12]. In particular, ewes selected for high litter size in abundant, energy-rich environments where they can satisfy their own needs and those of their litter may be at a high risk of health issues or reproductive failure when energy availability falls short. Taking an extreme example, ewes from wild sheep populations naturally selected in scarce environments tend to adopt a conservative pattern of energy allocation favoring their own survival and future reproduction rather those of their lambs when environmental conditions deteriorate [13]. So far, it is unclear to what extent energy allocation trade-offs between the ewe and her lambs (here defined as "ewe-lamb trade-offs") could underpin G × E interactions in ewe lifetime performance.

To address this issue, we developed a bioenergetic sheep model coupling the mother with her lamb(s) to study the effect of individual variation in energy acquisition and allocation on the performance of ewe and lamb [14]. Mathematical trade-off models represent relevant tools to explore by simulation the consequences of overall biological processes on animal responses to feeding conditions [5]. This allows the complex and dynamic interplay of physiological functions underlying life performance to be taken into account, albeit with an inevitable degree of simplification. Moreover, the fundamental assumption from the resource allocation theory that trade-offs reflect different genetic priorities of energy allocation can be integrated by considering individual variation in some model input parameters [15–18]. To date, trade-offs between mother and offspring have not been explicitly considered as most allocation models have focused on dairy animals. To explore if ewe-lamb trade-offs can lead to G × E interactions in lifetime performance, here we defined a broad range of “individual types” based on a relatively large set of input parameters that directly or indirectly control the energy acquisition and allocation of the ewe and her lambs. We simulated the lifetime performance of those individual types in two contrasting feed environments and hypothesized that (*i*) lifetime performance will be subject to G × E interactions, (*ii*) only a few input parameters will strongly and consistently influence lifetime performance in the two environments, (*iii*) ewe-lamb trade-offs assumed at the individual level will translate into negative correlations between ewe longevity and the production of lambs (i.e. prolificacy) or their survival among individuals.

## Methods

### Approach

**Fig. 1:**
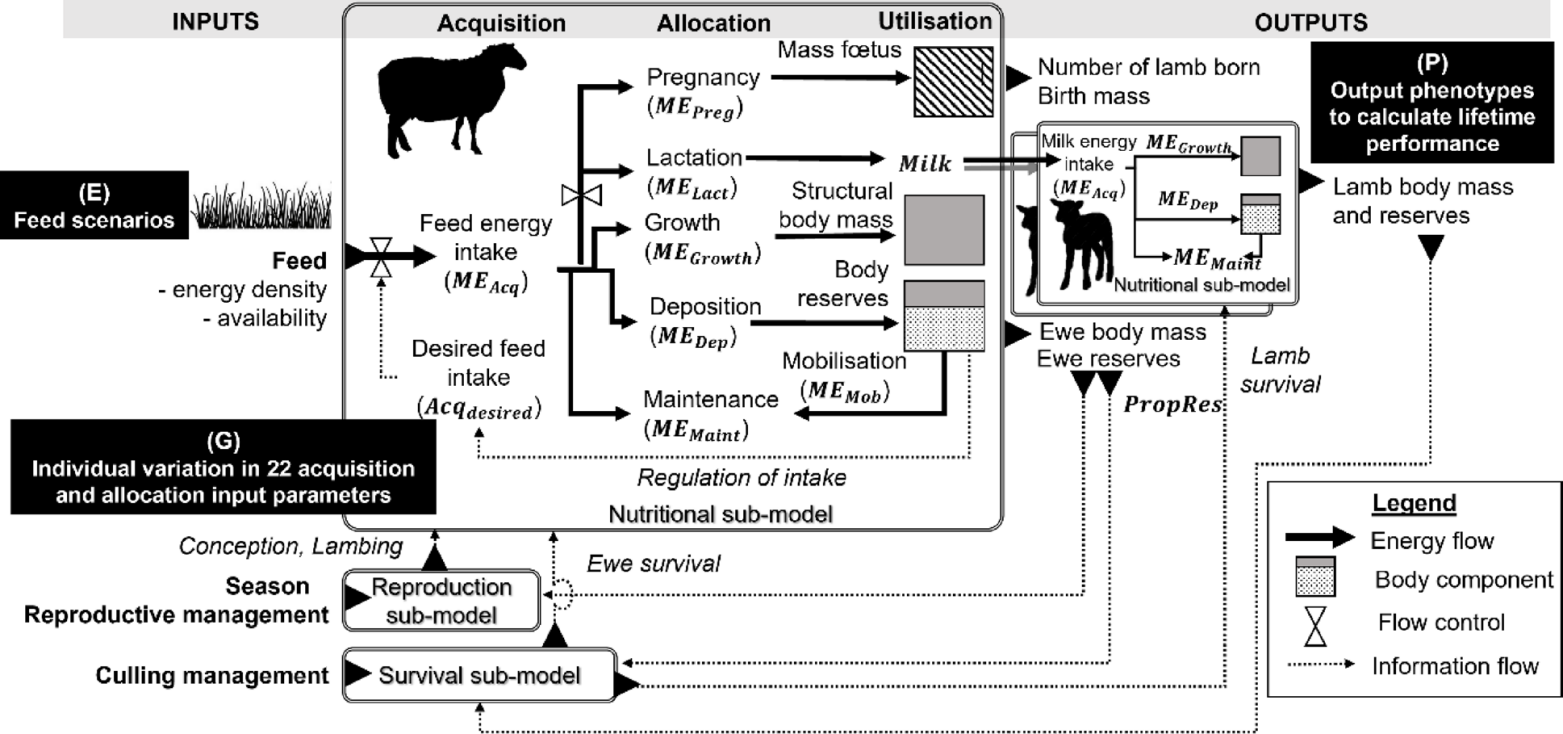
Conceptual diagram of the bioenergetic model linking the ewe to her lambs. The model was used to predict G × E interactions in the lifetime performance of the ewe. Outputs traits (“P”) are predicted according to the individual type (“G”; Table 1) and the feed environment (“E”; Fig. 2), both defined as inputs. Each component is detailed in the model description [14]

Our approach to explore G × E interactions in lifetime performance (Fig. 1) was based on a sensitivity analysis of our ewe-lamb energy allocation model [14] in two feed environments (“E” in Fig. 1). Based on the resource allocation theory, age-related patterns of animal performances are assumed to reflect the expression of genes controlling energy acquisition and allocation between life functions. In our model, 22 model input parameters associated with those mechanisms were preselected (“G” in Fig. 1 and Table 1 for parameter definition). Those input parameters were included in a sensitivity analysis to start exploring individual variation. Our analysis corresponds to the simplest case where (i) input parameters represent genetically and phenotypically independent biological traits and, (ii) the value of those traits closely related to the underlying biological processes remain constant across environments. For simplicity, we subsequently call an “individual type” any particular combination of the 22 input parameters. Given the high dimensional parameter space, we chose an efficient way of exploring it with a screening method for global sensitivity analysis (see details in 2.3.1 “Design of experiment”). As a result, a population of 2,760 individual types was sampled and simulated in the two feed environments. Outputs traits of each ewe type and her lambs (“P” in Fig. 1) were simulated using the individual based model described below from birth to a maximum of 11 years, if the ewe was still alive at that age, allowing the calculation of lifetime performance, in particular the total number of lambs weaned (TNLW) during her career. As our model assumed some stochasticity (i.e. in initial conditions at birth, and in survival and reproduction processes), we run 20 ewe replicates per individual type and per environment and reported the average values of outputs traits.

**Table 1:** Description of the 22 input parameters used to define each individual type and the range of values used in the sensitivity analysis with the Morris’ elementary effects screening method

Process	Input parameter	Description	Min	Max
Acquisition	${b}_{AcqStruct}$	Basal rate of feed acquisition (in kg of dry matter/day) per kg of structural mass driving the base level of energy acquisition (${ME}_{Acq}$) throughout life	0.23	0.28
	${PropRes}^{*}$	Constant target proportion of body reserves $PropRes$ (ratio of lipid to empty body mass) setting the regulation of intake throughout life; when the animal becomes relatively fat ($PropRes > {PropRes}^{*}$) then ${ME}_{Acq}$ is reduced, and vice versa	0.20	0.30
Growth	${MassStruct}^{*}$	Structural mass at maturity (in kg of protein in dry empty body mass) driving energy allocation to growth ($M{E}_{Growth}$) until structural mass ($MassStruct$) reaches ${MassStruct}^{*}$	6.00	7.00
	${AllocGrowth}^{*}$	Target allocation to growth (ratio of $M{E}_{Growth}$ to $M{E}_{Acq}$) at birth, driving growth rate in $MassStruct$	0.50	0.70
	${k}_{AllocGrowt{h}_{U}}$	Scaling exponent (unitless) controlling the exponential decrease in energy allocation to growth with physical maturity. High values of ${k}_{AllocGrowt{h}_{U}}$ promote a fast growth	0.20	0.33
Storage	${AllocResProt}^{*}$	Constant target allocation to protein reserves (ratio of lipid energy allocation to total reserves (lipid + protein) energy allocation). The quantity $(1-{AllocResProt}^{*})$ determines the deposition of lipid reserves throughout life	0.05	0.15
Sensitivity to body reserves variation	${b}_{AcqPropRes}$	Rate of change in feed acquisition (kg/day) per unit of body reserves ($PropRes$) deviation from ${PropRes}^{*}$ throughout life. High values of ${b}_{AcqPropRes}$ are associated with a high responsiveness of ${ME}_{Acq}$ to body reserves variation	0.10	0.30
	${b}_{AllocPropRes}$	Rate of change in energy allocation to pregnancy and lactation per unit of body reserves ($PropRes$) deviation from ${PropRes}^{*}$. High values of ${b}_{AllocPropRes}$ are associated with a high responsiveness to body reserves variation (e.g. when $PropRes< {PropRes}^{*}$ a fast decrease in allocation to pregnancy or lactation to favor ewe survival)	1.5	3.0
Pregnancy	${ovulrate}^{*}$	Innate ovulation rate (expected number of ovula produced) impacting the potential mass of the gravid uterus during pregnancy and the number of lambs at birth	1.2	3.2
	${b}_{AcqPreg}$	Rate of additional feed acquisition (in kg of dry matter/day) per kg of gravid uterus ($MassGU$), driving the level of ${ME}_{Acq}$ during pregnancy	0.02	0.08
	${AllocPreg}^{*}$	Target allocation to pregnancy (ratio of $M{E}_{Preg}$ to $M{E}_{Acq}$) at the end of the gestation period, driving $MassGU$ at lambing	0.40	0.70
	${k}_{AllocPre{g}_{time}}$	Scaling exponent (unitless) controlling the exponential increase in energy allocation to pregnancy with time of gestation. High values of ${k}_{{AllocPreg}_{time}}$ involves a fast growth of $MassGU$	2.5	4.0
	$pNL{1}_{Preg}$	Coefficients controlling the effect of the number of foetuses on energy allocation to pregnancy. The quantity $1/pNL{1}_{Preg}$ sets the extent of the maximum effect when ewes bear at least 3 foetuses while $pNL{2}_{Preg}$ define the percentage of this maximum effect for twin-bearing ewes	0.35	0.75
	$pNL{2}_{Preg}$		0.80	1.00
Lactation	${b}_{AcqLact}$	Coefficients controlling the rate of additional feed acquisition during lactation ($AcqLact$), then driving the level of ${ME}_{Acq}$ during lactation ${b}_{AcqLact}$ determines the overall magnitude of $AcqLact$, $k{1}_{AcqLact}$ its increase in early lactation and $k{2}_{AcqLact}$ its decrease from mid- to late lactation	0.50	1.30
	$k{1}_{AcqLact}$		0.025	0.045
	$k{2}_{AcqLact}$		0.020	0.050
	${AllocLact}^{*}$	Target allocation to lactation (ratio of $M{E}_{Lact}$ to $M{E}_{Acq}$) at lambing, driving milk production in early lactation	0.50	1.00
	${k}_{AllocLac{t}_{time}}$	Scaling exponent (unitless) controlling the exponential decrease in energy allocation to lactation with day of lactation. Low values of ${k}_{{Lact}_{time}}$ promote a high lactation persistency	0.0025	0.0100
	${k}_{AllocLac{t}_{U}}$	Scaling exponent (unitless) controlling the increase in energy allocation to lactation with physical maturity. High values of ${k}_{{AllocLact}_{U}}$ promote a high precocity in milk production	0.30	3.00
	$pNL{1}_{Lact}$	Coefficients controlling the effect of the number of suckling lambs on energy allocation to lactation. The quantity $1/pNL{1}_{Lact}$ sets the extent of the maximum effect when ewes suckle at least 3 lambs while $pNL{2}_{Lact}$ define the percentage of this maximum effect for ewes suckling twins	0.45	0.75
	$pNL{2}_{Lact}$		0.80	1.00

Input parameters marked with an asterisk indicate targets that an animal is assumed to seek

### Model overview

A complete, detailed model description, following the ODD (Overview, Design concepts and Details) protocol for individual-based models [19–21] is provided in [14]. The basic idea underlying the model is to use the energy allocation principle to predict the lifetime trajectories of trait responses of a suckler ewe and her lambs to feed energy availability and flock management practices. The ultimate model objective (not addressed here) will be to highlight the consequences of energy-allocation trade-offs assumed at the individual scale on genetic trade-offs among individuals, especially in populations under selection and in conditions that may occur in the future. To consider our model realistic enough for its current purpose, we checked patterns of lamb growth, ewe body mass and condition throughout successive production cycles.

The model includes a single entity: the individual sheep. It is characterized by three types of state variables: (1) physiological status and stage, (2) flows and stocks of matter or energy describing the transformation of feed into animal products and, (3) any dependency between the individual and another one (e.g. mother or lamb). As for the spatial and temporal resolution and extent: a time step in the model represents one day and simulations are run for a length defined by the input environmental time series. The model is not spatially explicit.

The most important processes of the model, which are repeated every time step, result in the update of animal’s state. A set of nutritional processes first predict the responses to current feed input (i.e. feed energy acquisition, allocation between life functions and utilisation). Part of those processes outputs (particularly body condition) along with management and environmental inputs are then used to subsequently evaluate reproduction and survival events so that animal’s state for the next time step is defined.

The most important design concept of the model is the assumption that energy allocation trade-offs between life functions underpins the lifetime trajectory of an animal from conception to death, that is a common assumption of many other bioenergetic models in animal science or in ecology (e.g. [22, 23]). A particular feature of our model is to explicitly represent the maternal energy transfers to the offspring and the potential competition among siblings if the total energy demand of the litter exceeds the amount of energy provided by the mother. Another key design concept is the consideration of individual variation in the model input parameters that directly or indirectly control energy allocation trade-offs, and which are used here to define “individual types”.

### Simulation

#### Design of experiment

To evaluate the relative importance of the 22 input parameter on the model outputs, we used the Morris’ elementary effects screening method [24] as implemented in the R package *sensitivity* [25]. For each input parameter ${X}_{k}$, we first defined a range ($min$ and $max$) (Table 1). This was done using a one-at-a-time local sensitivity analysis in which each parameter was varied independently between its lower and upper bounds while all other parameters were held at their default values. The bounds were chosen to generate sufficient variation in outputs directly related to bioenergetic process controlled by the parameters (e.g. feed intake, body mass, body condition) while remaining within a realistic biological order of magnitude (Additional file 1 Table S1). We then defined the number of levels $p$ giving the step $\Delta = 1/(p-1)$ between two successive values of scaled ${X}_{k}$ (here $p = 4$). The large *n*-dimensional *p*-level grid (here 4^22^ that is more than 10^13^ different combinations of parameter levels) is explored with the Morris method. For this, different nominal starting points are randomly sampled in the grid to initiate $r$ trajectories. In each trajectory, all parameter values are successively changed in a random order and one at a time by adding the step $\Delta$ (e.g. point 1 = $\left\{{X}_{1}, {X}_{2}, \dots ,{X}_{k}, \dots , {X}_{n}\right\}$, point 2 = $\left\{{X}_{1}, {X}_{2}, \dots ,{X}_{k}+ \Delta , \dots , {X}_{n}\right\}$). As a result, the number of combinations of parameter values explored is reduced to *r* · (*n* + 1). In our case, we chose $r=120$ so that 2,760 parameter combinations, here considered as different individual types, were explored. The exact same design was used in both feed scenarios to simulate outputs traits of each individual type in two contrasting environments. The other model input parameters were left constant, their values were based, where possible, on the available literature [14]. For simplicity, we assumed that lambs born during the simulation inherited the same 22 input parameter levels of their mother.

To analyze the simulated output traits, we performed a paired Wilcoxon signed rank sum test and we calculated Spearman correlations (φ) between the feed scenarios to check for differences in the performance.

#### Environmental scenarios

**Fig. 2:**
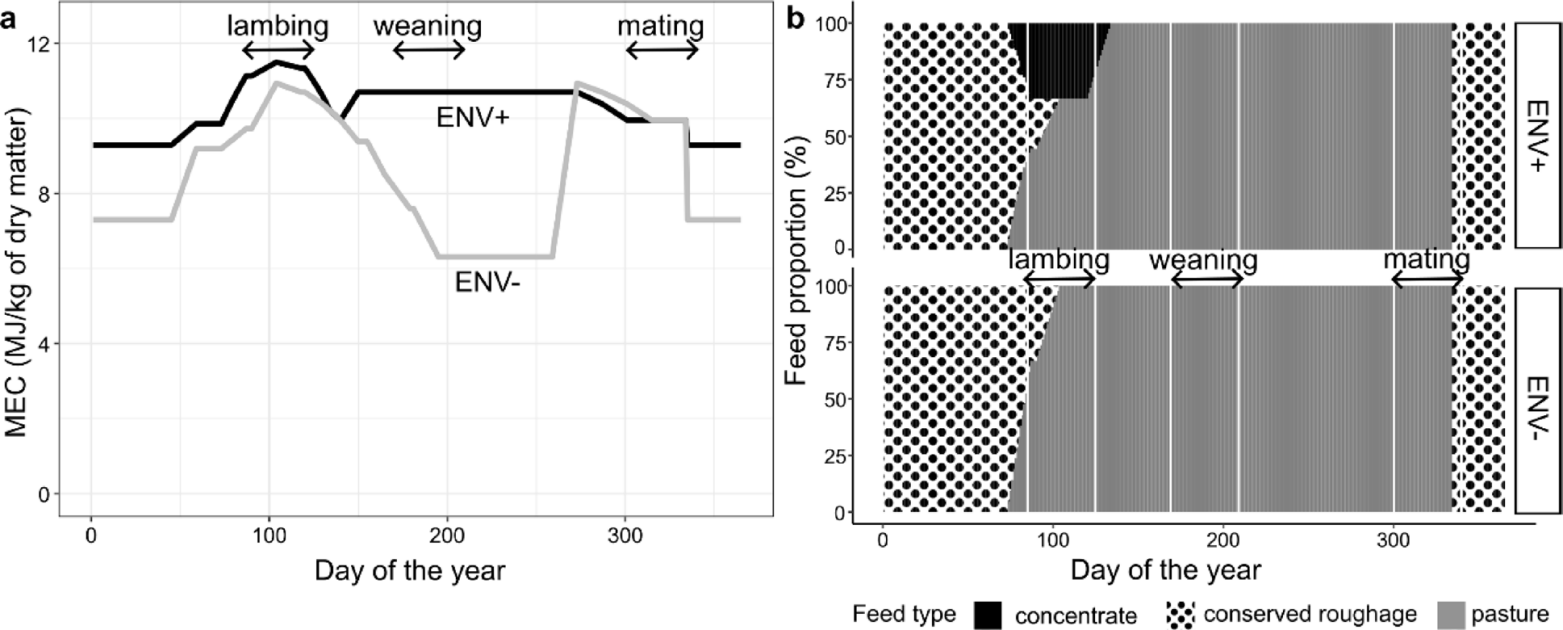
Feed scenarios and herd management. **a** Feed metabolizable energy content (MEC in MJ metabolizable energy / kg dry matter) for each day of the year in the favorable (ENV+) and the unfavorable (ENV-) environmental scenarios. The mating period in autumn leads to a lambing period in spring with the highest MEC from vegetative pasture. The suckling period started at lambing and ended with weaning in summer. **b** Relative proportions of pasture, concentrates and conserved roughage (hay and silage) simulated for each day of the year in the two environmental scenarios

Environmental scenarios were defined for a simulation time of 11 years, starting on January 1st of year 1. We defined a favorable (ENV+) and an unfavorable (ENV-) feed quality scenario by varying the metabolizable energy content (MEC in MJ/kg dry matter) of the feed over the year. The same intra-annual variations were assumed within a scenario (i.e. no interannual variation). Both scenarios reflect a pasture and roughage-based diet with a mixture of grasses and legumes in a middle European climate (latitude = 40°). All MEC values were obtained from the Swiss feed database (www.feedbase.ch). Feed proportions are shown in Fig. 2. Conserved feed (same roughages as grazed) was provided during winter feeding from December to mid-March, then from mid-March to begin April a gradual transition from conserved feed to pasture occurred, with grazing from April to end of November.

In ENV+ , there was a higher MEC in the feed than in ENV- (Fig. 2a), which included simulation of a summer drought period.

#### Initialization and termination

Simulations of the 2,760 individual types were replicated 20 times in each environment by randomly sampling different initial conditions regarding the day of birth (between day 1 and day 30 of simulation) and mass at birth to mimic natural variation within a herd contemporary group. All replicates survived at birth. The minimal age for mating ewe lambs was set to 8 months (240 days). The mating period was defined from day 300 to day 340 of the year (end of October to start of December). Ewes lambed accordingly after a gestation period of 150 days between day 85 to day 125 of the year (end of March to start of May). After a suckling period of 3 months (84 days), all lambs (males and females) were weaned and removed from the simulation. The initial ewes were not culled even if they failed to reproduce. They lived until death or until the end of simulation otherwise (maximum of 11 years). Ewe death occurred randomly based on a mortality risk function controlled by age and body reserves.

#### Model outputs

Outputs traits of each individual type in a given environment were evaluated based on the average lifetime performance of the 20 replicates, including animals that died before the end of the simulation. Lifetime performance consisted of the total number of lambs born (TNLB), born alive (TNLBA), and weaned per ewe (TNLW). Ewe longevity was described by the mean and median number of parities, the age at death and the number of ewes still alive at each year of simulation. A number of within parity averages were also calculated: lamb birth traits are the lamb birth mass, the number of lambs born (NLB) and born alive (NLBA) and the proportion of lambs born dead for the parities 1, 2 and 3 and following. Lamb survival traits are the proportion of lambs surviving from birth to 42 days and to 84 days (weaning). Lamb growth traits are the daily growth from birth to day 42 and 84 and body mass at 42 and 84 days respectively. Ewe outputs include mean ewe body mass, body fat reserve level, food intake, and food energy intake at the start of the mating period for each parity. The same traits, as well milk yield, were recorded in lactating ewes at parturition, mid-lactation and at weaning for each parity.

#### Sensitivity indices

We calculated the Morris indices µ* and σ for each one of the *n* input parameters in both feed scenarios. For each input ${X}_{k}$ whose value is changed by $\Delta$, the elementary effect ${EE}_{k}$ is defined for a given model output $Y$ as:

$$ EE_{k} = \frac{\begin{gathered} Y(X_{1} ,X_{2} , \ldots ,X_{k} + \Delta , \ldots ,X_{n} ) \hfill \\ \quad \quad - Y(X_{1} ,X_{2} , \ldots ,X_{k} , \ldots ,X_{n} ) \hfill \\ \end{gathered} }{\Delta } $$

Absolute values of ${EE}_{k}$ are averaged over the *r* repetitions to calculate ${\mu }_{k}^{*}$ reflecting the degree of influence of ${X}_{k}$ on $Y$.

$${\mu }_{k}^{*} = \frac{1}{r}{\sum }_{j = 1}^{r}|{EE}_{k}^{(j)}|$$

The standard deviation ${\sigma }_{k}$ is also calculated based on the *r* values of ${EE}_{k}$ to evaluate the degree of non-linearity of the effect (increasing with ${\sigma }_{k}$) and its potential interactions with other parameters.

To check the uncertainty of the statistics, we used a bootstrap approach to calculate µ* and σ from a subset of 24 repetitions each which gave a range of values.

## Results

### Lifetime performance

**Fig. 3:**
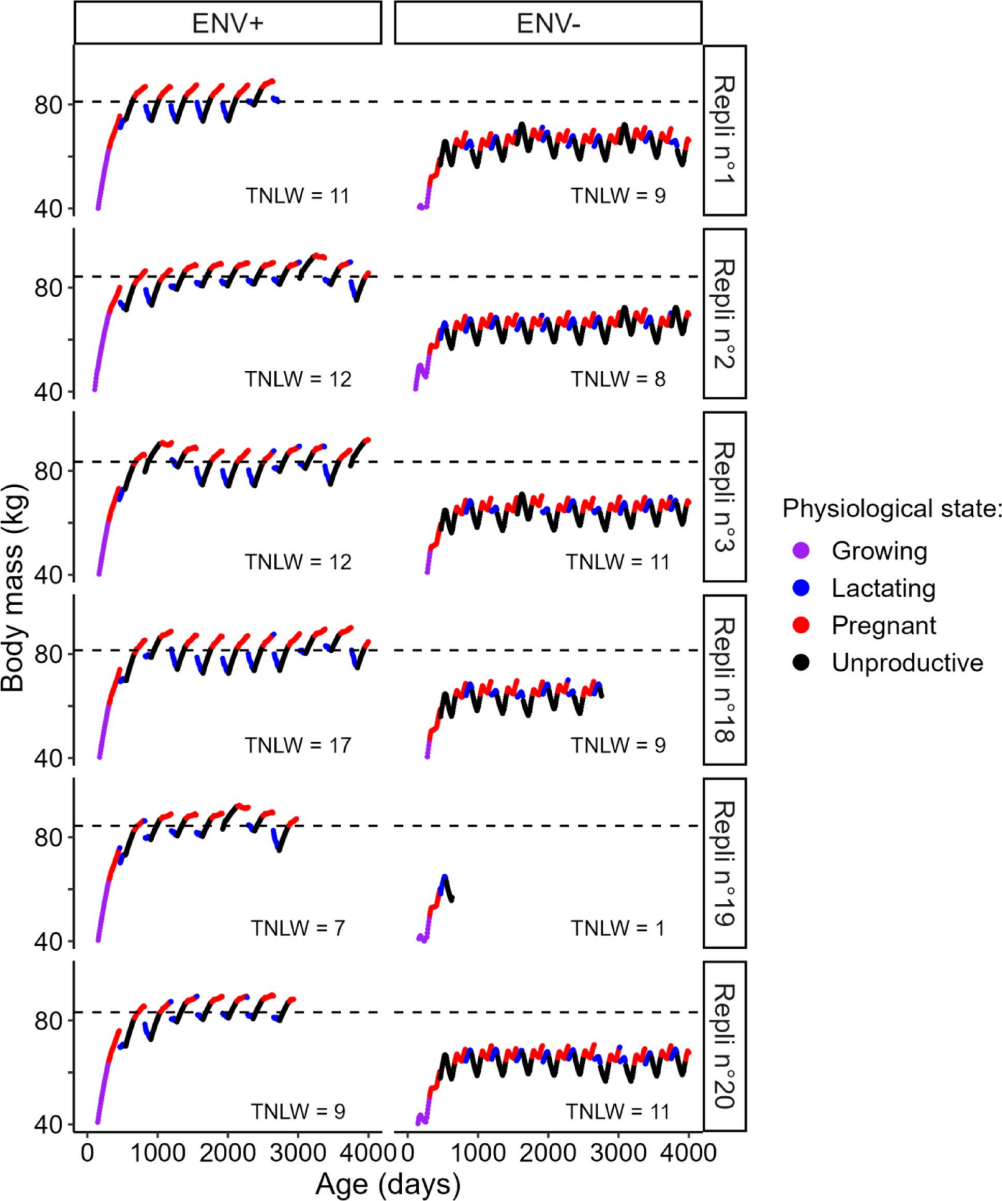
Illustration of lifetime trajectories of body mass for six replicates of a virtual individual type in favorable (ENV+) and unfavorable (ENV-) feed environments, with the corresponding lifetime performance approximated by the total number of lambs weaned (TNLW). The individual type corresponds to a pattern of energy allocation defined by a unique combination of the 22 input parameter of Table 1. The individual type chosen among the 2,760 simulated ones, has the mean values of TNLW closest to the means of all individual types in the two environments (TNLW = 9.95 and 5.55 in ENV+ and ENV-, respectively). Only 6 of the 20 replicates are shown here

Model outputs are first illustrated with a few replicated lifetime trajectories of a particular individual type (Fig. 3). The feeding environment clearly and consistently affected traits directly determined by bioenergetic processes such as adult body mass (e.g. for the individual type shown in Fig. 3, mean and SD of all replicates (n = 20): 83.1 and 1.07 kg in ENV+ vs. 64.1 and 1.99 kg in ENV-). At the population level, output traits directly related to those processes were in a realistic range (e.g. mean [min; max] for adult body mass at mating: 72.7 kg [52.9; 111.9] in ENV+, 60.6 kg [47.5; 78.3] in ENV-; for ewe feed intake at mid-lactation: 1.99 kg/day [1.34; 3.29] in ENV+ , 2.09 kg/day [1.44; 3.40] in ENV-).

**Fig. 4:**
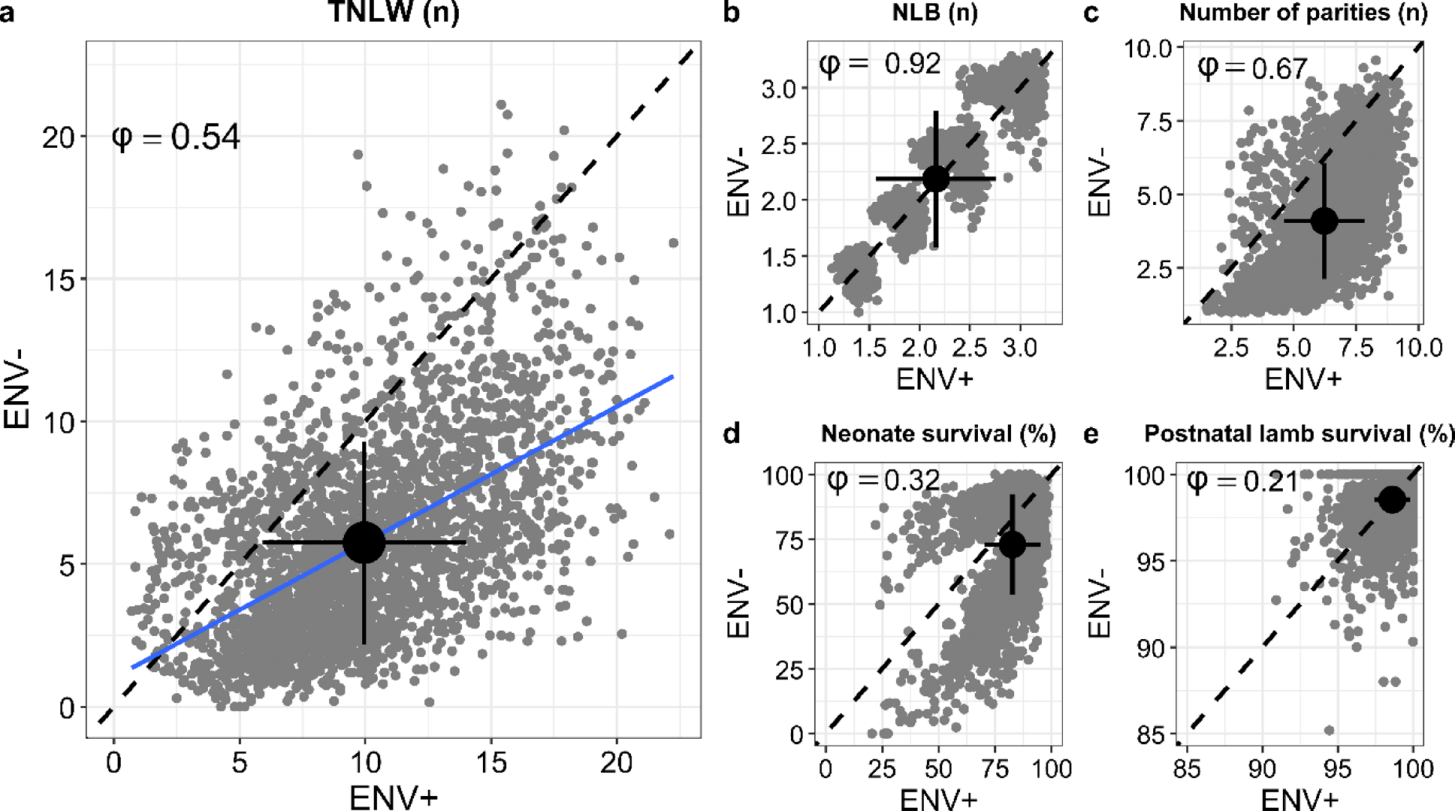
Relationship between ewe outputs traits in favorable (ENV+ ; x-axis) and unfavorable (ENV-; y-axis) environments. Output traits include **a** total number of lambs weaned per ewe = TNLW, **b** litter size at birth = NLB, **c** number of parities per ewe, **d** neonatal survival and **e** postnatal lamb survival until weaning. Each grey point (n = 2,760) represents an average value of individual type (20 replicates per individual type), where each individual type corresponds to a particular combination of the 22 input parameters (Table 1). Black points with error bars represent the overall means with 95% confidence intervals. Dashed line represents the line of equality between the two axes. Blue line in (a) indicate the relationship of linear regression

Lifetime performance, approximated in this study by the total number of lambs weaned (TNLW), was more variable among replicates due to random variation in survival and reproductive processes. However, TNLW of the 2,760 individual types was reduced in ENV- compared with ENV+ (Table 1), with an average reduction of 36.6% (95% confidence interval: [34.7%, 38.5%]. In line with expectation (*i*), we found a moderate Spearman correlation of TNLW between the two environments (φ = 0.54, Fig. 4a). Pearson correlation was close (*r* = 0.53). In the remaining of the Results, only mean trait values calculated across replicates within individual type and within environment are considered.

### Components of lifetime performance across environments

TNLW was mainly determined by prolificacy (NLB), neonatal survival, postnatal survival and ewe longevity (Fig. 4b-e). Lamb survival and ewe longevity were significantly lower in ENV- than in ENV+ whereas NLB remained almost unchanged (Table 2). The correlation of NLB between environments was high (φ = 0.92, Fig. 4b). The lower feed quality during pregnancy in ENV- led to a decrease of about 28% in lamb birth mass compared with ENV+, and consequently, to a strong decrease in neonatal survival (73.2% survival vs. 82.7%). Other factors reducing lamb birth mass (e.g. high NLB, low ewe parity) also led to reduced neonatal survival. Contrary to NLB, neonate survival was only moderately correlated between environments (φ = 0.33) (Fig. 4d, Table 2). Some individual types showed lower neonate survival in ENV+ than in ENV-, as energy-rich conditions led to higher birth mass and thus a greater risk of dystocia, while poorer conditions produced lighter neonates with a reduced risk. Postnatal lamb survival was generally high (~ 96 – 99%), yet relatively variable among individual types in ENV- and poorly correlated between environments (φ = 0.17, Fig. 4e). The number of parities per ewe was moderately correlated between environments (φ = 0.63, Table 2, Fig. 4c).

**Table 2:** Mean of outputs traits (with SD) in favorable (ENV+) and unfavorable (ENV-) environments and Spearman correlations between environments (φ)

Trait	Unit	ENV+		ENV-		φ
		Mean	SD	Mean	SD	
TNLW	n	9.95	4.04	5.75	3.55	0.54
TNLB	n	12.16	4.68	8.19	4.77	0.69
Number of parities	n	6.23	1.63	4.10	1.97	0.67
Number of ewes alive at 11 years (out of 20 replicates)	n	6.92	4.18	3.13	3.44	0.49
NLB	n	2.16	0.59	2.19	0.61	0.92
NLW (including ewe who lost all lambs)	n	1.76	0.52	1.49	0.52	0.67
Neonate survival	%	82.7	12.3	72.9	19.3	0.32
Postnatal lamb survival	%	98.6	1.2	98.5	4.1	0.21
Lamb mass at birth	kg	4.39	1.41	3.17	1.18	0.93
Lamb mass at 42 days	kg	15.64	3.64	13.03	3.12	0.94
Lamb mass at weaning	kg	28.23	6.04	23.09	5.04	0.95
Lamb daily growth from birth to 42 days	g/day	269	70	232	60	0.94
Lamb daily growth from birth to weaning	g/day	284	64	236	54	0.96

Sample size include 2,760 ewe individual types evaluated in the two environments (each individual type output was the average of 20 ewes replicates and their lambs). One individual type corresponds to a particular combination of the 22 input parameters (see Table 1). The values are calculated from all animals in the corresponding parity. All values of φ were significantly different from 0 (*p* < 0.001; paired Wilcoxon rank sum test)

NLB = Number of lambs born, NLW = number of lambs weaned, TNLW = Total NLW, TNLB = Total NLB

Finally, correlations between TNLW and its main components varied across environments. In ENV+ , TNLW was strongly correlated with prolificacy (i.e. with NLB: φ = 0.64) and ewe longevity (i.e. with number of parities: φ = 0.61) whereas in ENV-, TNLW was only moderately correlated with NLB (φ = 0.31) and more strongly with longevity (φ = 0.79).

### Sensitivity of ewe lifetime performance to input parameters

**Fig. 5:**
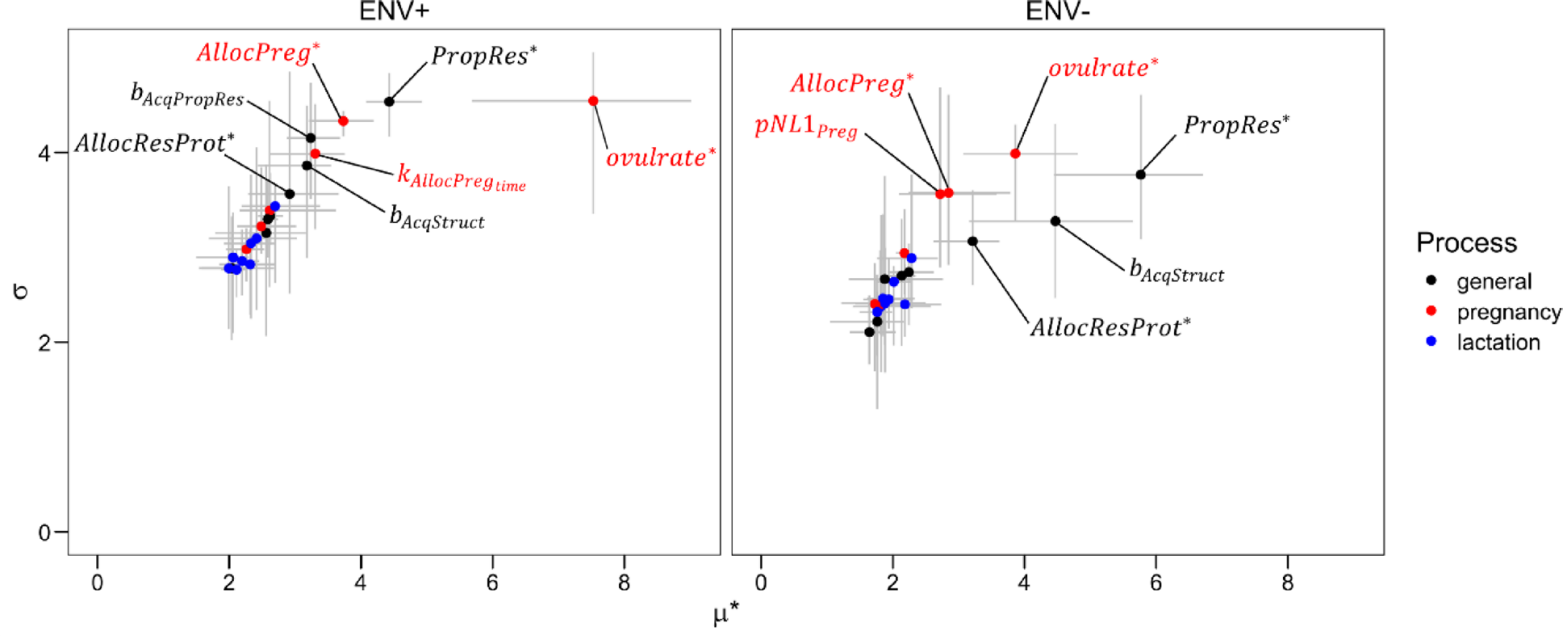
Effect size estimates of varying the 22 input parameters on total number of lamb weaned (TNLW) including linear effects (µ*) and non-linear and interaction effects (σ). Parameters are classified as general (black), or related to pregnancy (red), or lactation (blue) (see Table 1 for parameter definition). The greater the values the more influence that parameter has on TNLW. Estimations of effect size are based on 120 repetitions in the favorable (ENV+) and the unfavorable (ENV-) environment with the Morris' elementary effects screening method. The whiskers show the upper and lower bounds of the uncertainty on µ* and σ calculated from 5 times 24 repetitions based on subsampling of the 120 repetitions

**Fig. 6:**
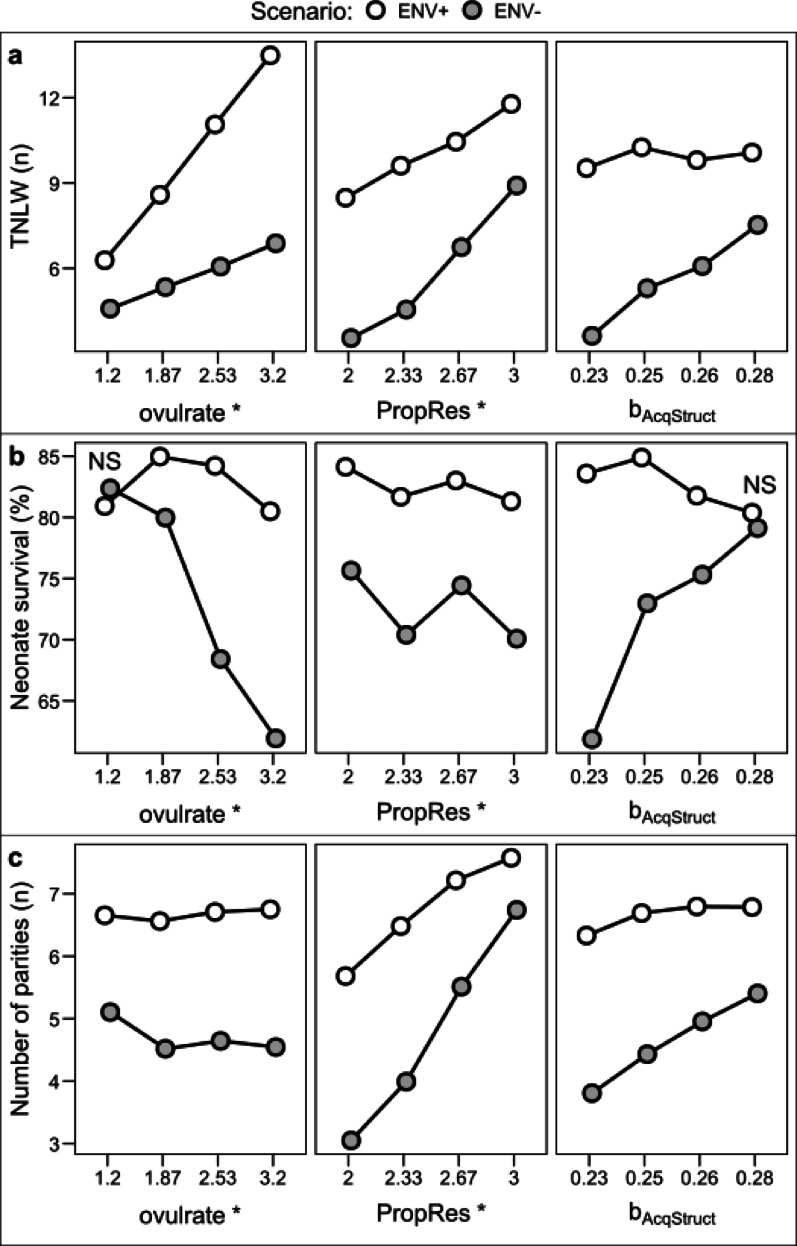
Effects of the three most influencing model input parameters on outputs traits. Output traits include **a** total number of lambs weaned (TNLW), **b** neonate survival and **c** ewe longevity. Input parameters include the target ovulation rate ($${ovulrate}^{*}$$), the target fat reserve proportion ($${PropRes}^{*})$$ and basal feed intake ($${b}_{AcqStruct}$$) (See Table 1 for definition). Each point represents the mean of about 690 individual types (min = 557, max = 814). Relative standard errors of the means were between 0.4% and 2.8% (1.2% on average). Pairwise comparisons between environments were all significant, except those marked with NS

As we expected (prediction *ii*), the effect screening of the 22 varied input parameters (Table 1) showed that a few input parameters strongly influenced TNLW in both environments (Fig. 5 and Additional file 2 Table S2 for all values of μ* and σ). This included prolificacy potential (${ovulrate}^{*}$), and two parameters related to energy acquisition target: fat reserve proportion (${PropRes}^{*}$), and basal intake rate (${b}_{AcqStruct}$). In both environments, ${ovulrate}^{*}$ affected TNLW mainly through a linear effect on NLB (see the four clusters corresponding to the four levels of ${ovulrate}^{*}$ in Fig. 4b). However, increasing levels of NLB driven by ${ovulrate}^{*}$ decreased lamb birth mass and neonate survival as a consequence, especially in ENV- (Fig. 6a). As a result, the overall effect of ${ovulrate}^{*}$ on TNLW was strongly limited in ENV- compared with ENV+ (Fig. 6a).

In ENV-, TNLW was more sensitive to ${PropRes}^{*}$ (Fig. 6b) and ${b}_{AcqStruct}$ (Fig. 6c). The influence of ${PropRes}^{*}$ on TNLW was mainly through ewe longevity (ENV+ : µ* = 2.61 parities and σ = 2.18 parities, ENV-: µ* = 4.10 and σ = 1.79). Ewes with higher levels of ${PropRes}^{*}$ lived longer, especially in ENV- (Fig. 6c). Finally, increasing levels of energy acquisition ${b}_{AcqStruct}$ led to increased TNLW through both higher neonate survival (Fig. 6b) and through higher ewe longevity (Fig. 6c), especially in ENV-.

### Ewe-lamb trade-offs

**Fig. 7:**
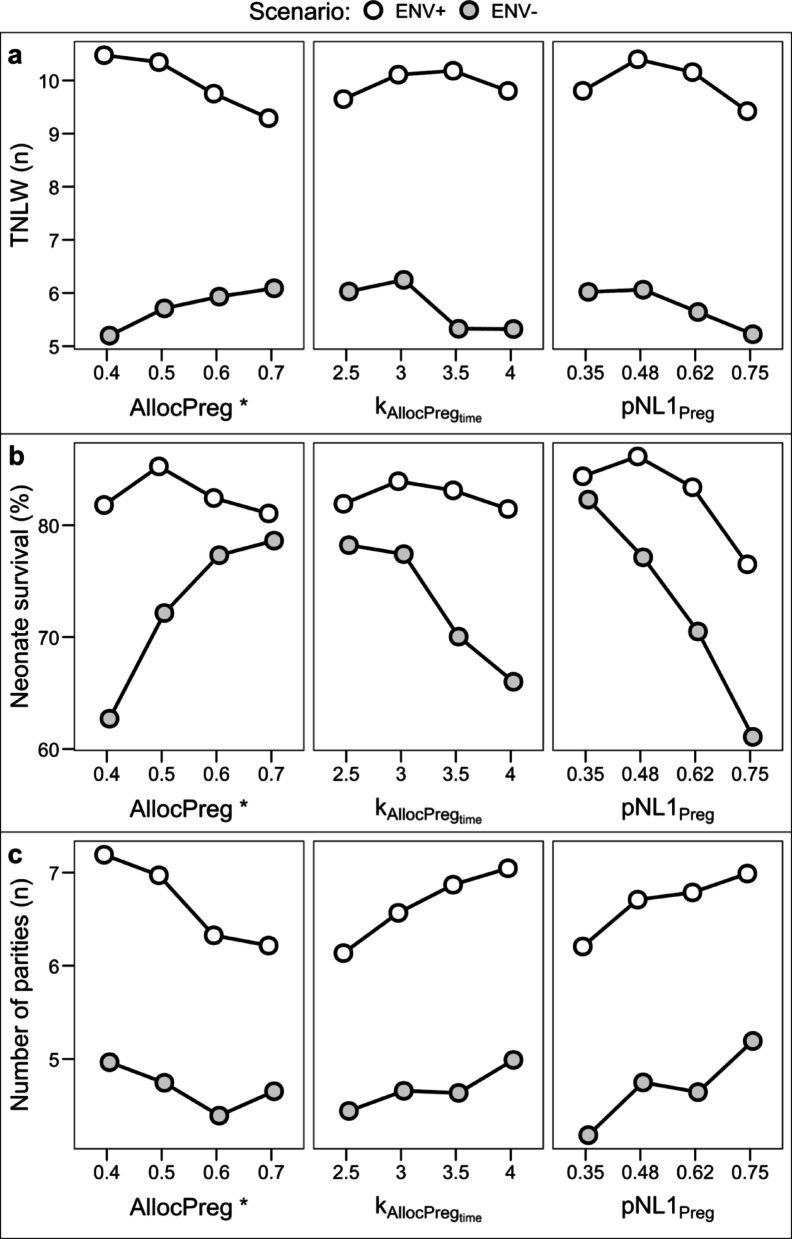
Effects of three input parameters controlling allocation to pregnancy on output traits. Output traits include **a** total number of lambs weaned (TNLW), **b** neonate survival and **c** ewe longevity. Input parameters include target allocation to pregnancy (${AllocPreg}^{*}$), the change in allocation to pregnancy with gestation time (${k}_{AllocPre{g}_{time}}$) and with litter size ($pNL{1}_{Preg}$) (See Table 1 for definition). Each point represents the mean of about 690 individual types (min = 585, max = 820). Relative standard errors of the means were between 0.4% and 2.3% (1.3% on average). Pairwise comparisons between environments were all significant

Contrary to our expectation (*iii*) that ewe-lamb trade-offs assumed at the individual level would lead to negative correlations between ewe longevity and lamb components of TNLW (NLB or neonate survival) among-individuals, those were close to zero in both environments. Moreover, the three influencing parameters previously highlighted (Fig. 6) did not affect TNLW components in opposite directions. However, our results confirmed that several input parameters controlling energy allocation to pregnancy or lactation directly mediated ewe-lamb trade-offs (Fig. 7). This included the target allocation to pregnancy (${AllocPreg}^{*}$) and its modulation with days of gestation (${k}_{AllocPre{g}_{time}}$) or with litter size ($pNL{1}_{Preg}$). Those parameters had a moderate influence on TNLW (Additional File 2 Table S2). However, higher levels of energy allocation to pregnancy (through an increase in ${AllocPreg}^{*}$ or in $pNL{1}_{Preg}$ or through a decrease in ${k}_{AllocPre{g}_{time}}$) led to higher neonate survival (Fig. 7b) but also to a reduction in ewe survival (Fig. 7c). A similar effect was observed for energy allocation lactation that favored lamb growth at the expense of ewe survival (${AllocLact}^{*}$, not shown).

### Characterization of robust ewes

**Fig. 8:**
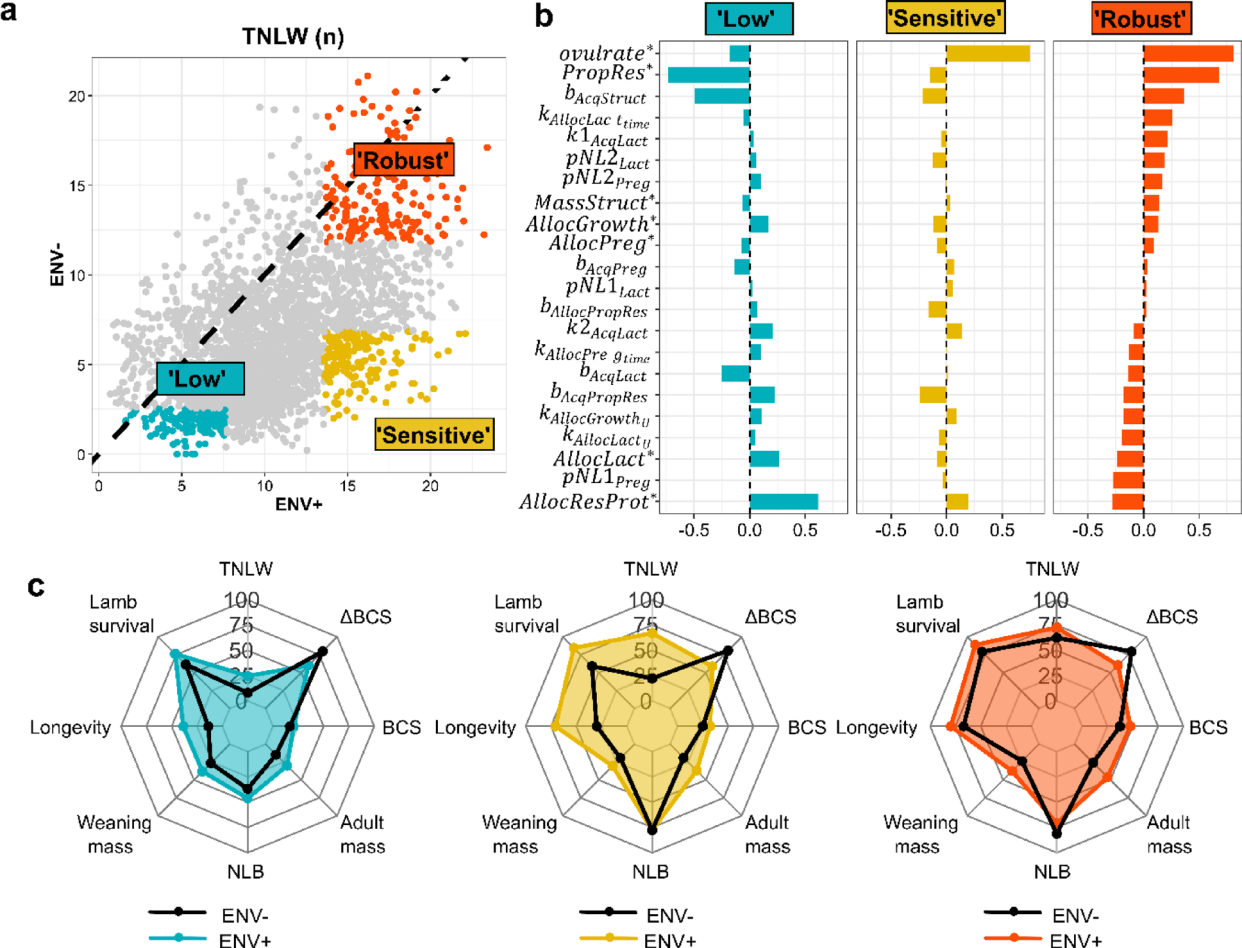
Extreme ewes regarding their lifetime performance in favorable (ENV+) and unfavorable (ENV-) environments. Each one of the three extreme groups represents 1/16th of the individual types defined according to their total number of lambs weaned (TNLW), with **a** “Low” = first ENV- quartile within the first ENV+ quartile; "Sensitive" and "Robust" = first and last ENV- quartiles within the last ENV+ quartile. The dashed line represents the line of equality between the two axes. **b** Average values of input parameters within each group (after rescaling between -1 and 1; see Table 1 for parameter definition). **c** Average standardized values of output traits in ENV+ and ENV- environments. BCS = body condition score at mating in 3^rd^ parity. ΔBCS = difference between BCS at weaning and BCS at mating in 3rd parity. NLB = mean number of lambs born

Based on the relationship of TNLW between environments (Fig. 4a), we characterized three extreme individual types (Fig. 8a). As previously observed (Fig. 5 and Fig. 6a), a high potential ovulation rate mainly distinguished the most productive ewes from the least productive individual types ("Low") in the favorable environment (Fig. 8b). Within the most productive ewes in ENV+ we identified two individual types "Sensitive" and "Robust" based on their performance in ENV-. In contrast to "Sensitive" ewes, "Robust" ewes generally combined a high ${ovulrate}^{*}$ with a high target body condition, which allowed them to achieve a high TNLW in both environments. "Robust" ewes were generally characterized by a low allocation to protein reserves (${AllocResProt}^{*}$) and a thus a higher allocation to fat reserves compared with both "Low" and "Sensitive" ewes. Low allocation to protein reserves increased neonatal survival and ewe longevity, as lower protein mass decreased the energy maintenance costs. Together with the lower allocation to lactation and the lower milk production for the lambs, "Robust" ewes weaned many, but lighter lambs. Compared with other ewes, "Robust" ewes had high values in a range of output traits and in both environments (Fig. 8c), except weaning mass in general and adult mass in ENV+.

## Discussion

Our bioenergetic mechanistic model predicted the responses of a range of virtual ewe individual types to two grassland-based feed environments that contrasted in terms of feed energy density. We focused on ewe lifetime performance, defined as the total number of lambs weaned (TNLW) during the entire life, as the environmental sensitivity of this trait can serve as a proxy for actual robustness. We found that TNLW was globally sensitive to feed energy availability and that this sensitivity varied among individual types. This brought support to our initial hypothesis (*i*) that an interaction between ewe types and their environment contributes to lifetime performance (φ = 0.54), suggesting individual variation in ewe robustness.

As in other mechanistic modelling studies [26–28], we considered individual variation at the level of biological mechanisms that are genetically determined. In this framework, the underlying traits (here described with the input parameters of an individual type) are assumed to be close to the biology so that their value remains constant across environments [27]. Although this is clearly a simplifying assumption, our approach can be viewed as a first test of the hypothesis that energy allocation processes can explain an important part of the phenotypic variation usually observed in various outputs traits (e.g. lifetime performance, ewe longevity, lamb survival). While the 22 underlying traits considered in this study had direct consequences on bioenergetic outputs (i.e. feed intake, body mass and condition), only a few of those underlying traits influenced ewe robustness.

Indeed, we found that different energy allocation patterns can correspond to differences in ewe robustness (Fig. 8). More precisely, sensitive ewes exposed to poor feeding conditions face energy allocation trade-offs between somatic functions and the production of lamb mass (mainly determined by the parameter ${ovulrate}^{*}$), which leads to a high risk of death, in particular for their litter. In contrast, robust ewes characterized by high prolificacy (${ovulrate}^{*}$), but also high target of body reserves (${propRes}^{*}$) or high intake capacity (${b}_{AcqStruct}$) manage well the consequences of ewe-lamb trade-offs and successfully wean many lambs, even under poor feeding conditions. This suggests that ewe-lamb trade-offs assumed at the individual level do not necessarily limit lifetime performance among individuals when feeding conditions deteriorate. Still, our simulation results suggest that TNLW is a partial proxy of robustness as some negative consequences on lactation traits, and subsequently on lamb body mass, were observed. Moreover, a high intake capacity was not detrimental in our unfavorable scenario —where feeding limitations were assumed in terms of quality rather than quantity— but it could become disadvantageous under real‑world situations where feed availability is limited. In such contexts, animals with high intake requirements may be disproportionately affected by extrinsic constraints. It is also important to note that our characterization of robust ewes relies on a modelling framework that focuses on energy dynamics. Consequently, the importance of biological traits that are critical for coping with non-energetic constraints may have been underestimated. For instance, although our results suggest that robust ewes store less proteins and more lipids than other ewes, protein reserves also play key roles in robustness, particularly to support immune function (e.g. [29]). Overall, our study highlights potentially important attributes of ewe robustness, but those findings now require validation against empirical evidence.

In addition, our findings rely on several key assumptions. In particular, certain individual types assumed in this study may actually not exist if at least some of our underlying traits turn out to be not biologically independent. For instance, if there was a strong negative genetic correlation between ${ovulrate}^{*}$ and ${propRes}^{*}$ this may preclude the existence of the “robust” ewe types assumed in our design. More generally, we did not account for biological mechanisms underlying trade-offs beyond energy allocation constraints, and these could lead to different results [30]. Besides biological factors, the covariances between underlying biological traits may also be shaped by ongoing selection. Likewise, management factors could also limit the presence of certain individual types. For instance, unproductive ewes (e.g. “low” ewe in Fig. 8) are unlikely to be maintained in managed flocks. If culling management was implemented in our study, most ewes would not live until their “natural death” or until the end of the simulation. For instance, simulating the culling of ewes that failed to reproduce led to a decrease of about 30% in longevity and TNLW in ENV+ and about 50% in ENV-, but the correlations among outputs traits were not substantially modified (Additional file 3 Table S3). Still, a more realistic simulation of ewe longevity accounting for the main culling reasons might be needed to further investigate the consequences of ewe-lamb trade-offs in farm conditions.

Looking at the components of TNLW, actual prolificacy (NLB) clearly had a main and generally positive influence. In contrast to TNLW, NLB was however relatively independent from the environment (φ = 0.92). This independence may seem inconsistent given that the heritability of NLB is generally around 0.1 [31]. However, our simulation of NLB was based on an expected rate of ovulation (i.e. ${ovulrate}^{*}$) but also on a variability around this level within-individual (assumed to be constant). Accordingly, the four clusters in Fig. 4b should rather be viewed as various prolificacy potentials among breeds rather than a variation within a single breed. Accordingly, within each cluster the correlation of NLB between environments was around 0.2.

Given the relatively large variation in litter size simulated in both environments, a negative influence of ewe prolificacy on TNLW could arise as a consequence of ewe-lamb trade-offs on lamb survival and on ewe longevity. However, this effect was only confirmed for lamb survival at birth. Such trade-off between offspring quality and quantity is ubiquitous among polytocous species [32, 33]. Here, poor survival was mediated by low birth mass, that is indeed a primary risk factor in sheep [34, 35]. In particular, many prolific ewes lost their lambs at lambing when feed resources were scarce, which suggests that maternal energy expenditure in late gestation (rather than during lactation) was the first limiting factor in our simulations. In contrast, prolificacy and ewe longevity were apparently independent, even in the unfavorable scenario. Similarly, ewe longevity was not affected by increased twinning rate in Scottish Blackface ewes [36]. In other studies, ewes that consistently express a high genetic potential for prolificacy (e.g. triplets) in the first parities were at higher risk of mortality than those with a moderate potential (e.g. twins) [12, 37, 38]. Such carry-over effects of high litter size could be mediated by an excessive loss of body reserves during the production cycle. However, this hypothesis did not seem to be supported here, possibly because the feeding level in autumn or the absence of culling management allowed heavily depleted ewes to recover or to remain unproductive.

Our study design allowed a first screening of the model sensitivity to a large number of input parameters. The choice of the input parameter range and the level of the parameters that were assumed constant is critical for the outcome of such a sensitivity analysis. In addition, at least three limitations existed with the chosen design. First, input parameters were assumed independent and uniformly distributed. This means that each level of parameter level could be combined with any other level of the other parameters, even though it is unclear if all combinations can exist or if the input parameters might be correlated. Moreover, uniform sampling probably led to an overrepresentation of extreme parameter values in our parameter space, which may have inflated the magnitude of correlations among outputs compared with what would be expected under a realistic multivariate normal distribution. Second, ewes gave birth to lambs with the same input parameters. Therefore, the lambs were assumed to be the clones of their mothers. Introducing genetic variance between ewes and lambs and the inheritance of input parameters from mother and father would enable to simulate a sheep herd over multiple generations. This might also allow to simulate crossbreeding systems with terminal sires on maternal lines for meat lamb production. A third limitation indeed, was to simulate a single cohort without any parental relationship between the pseudo genotypes. To go beyond those limitations, a simulation approach at the population level would be needed although it would require to focus on a smaller subset of influential parameters, in particular to calibrate the model. In this perspective, this study provides a first important step.

Based on a mechanistic model, it is indeed possible to simulate individual variation in the model input parameters to explore G × E interactions [17, 39]. In general, the parameters of interest are supposed to reflect a genetic effect (independent from the environment) and a permanent environmental effect [26, 27]. Our exploratory approach differ from previous works that used resource allocation models and that directly assumed individual variation in a few input parameters (generally less than 5) among the numerous model parameters (several tens) [15, 16, 27]. The choice of those parameters is usually based on their biological relevance regarding the focus of the study. Here, the most relevant parameters for lifetime performance were not necessarily obvious to choose a priori since a particular model parameter can affect multiple model outputs related to the ewe and her lamb(s) and interact with other parameters. Here we thus illustrated an approach to identify a subset of model parameters that will be relevant to further explore G × E interactions in lifetime performance measured as the total number of lambs weaned per ewe, and that can complement the choice of biologically relevant model parameters. It is important to keep in mind that the most influential parameters might differ when the focus is on other traits.

Considering the future environment is useful to adjust the breeding goal for today’s selection to obtain robust future animals. The environment definition might include the farming system, the feed, societal, ecological and economical expectations. The expected performance involves multiple traits. A mechanistic model predicting the animal’s traits in a given environment using the principle of energy allocation can be useful to shed light on the processes in the animal leading to those phenotypes. This might help to assess the occurrence of undesired side-effects commonly associated with selection for high production efficiency [40]. The resource allocation framework has an explicative and predictive power of the genotype’s performance in varied environments, and thus can help to understand why G × E interactions occur.

## Conclusion

Here we presented the first bioenergetic model, to our knowledge, that accounts for the consequences of energy trade-offs between the mother and her offspring on animal robustness. Specifically, we explored the consequences of ewe-lamb energy allocation trade-offs on maternal lifetime performance in contrasting feed environments. We found that lifetime performance was only moderately correlated between environments, which is consistent with a significant G × E interaction. This interaction was mainly mediated by a reduction in ewe and lamb survival under scarce resource conditions but those consequences were not directly caused by ewe-lamb trade-offs. This outcome illustrates the complexity arising from considering individual variation in a relatively large number of underlying biological traits controlling energy acquisition and allocation. As we identified some individual types that can successfully manage the consequences of ewe-lamb trade-offs, our study may provide insights for the selection of robust genotypes.

## Supplementary Information

Below is the link to the electronic supplementary material.

Supplementary Material 1
*Format*: docx. *Title*: Influence of input‑parameter ranges on model outputs associated with their underlying bioenergetic processes. *Description*: A table summarizing the local effect of varying each input parameter within its defined range on a selected model output. For each parameter, the selected output is associated with a bioenergetic process that the parameter directly controls (even though parameters often control several processes). Parameters were varied independently between their lower and upper bounds while all others were kept at default values. Simulations were performed in the favorable environment. Details on parameters definitions and environmental scenarios are provided in Materials & Methods.

Supplementary Material 2
*Format*: docx. *Title*: Table S2 Sensitivity indices of lifetime performance, neonate survival and ewe longevity. *Description*: A table reporting effect size estimates of varying the 22 input parameters on total number of lambs weaned (TNLW), neonate survival, and ewe longevity in the favorable (ENV +) and unfavorable (ENV-) environments. The 22 input parameters are defined in Table 1. Estimates of the sensitivity indices include linear effects (µ*) and non-linear and interaction effects (σ). The greater the values the more influence that parameter has on output traits. Estimations of effect size are based on 120 repetitions in each environment with the Morris's elementary effects screening method.

Supplementary Material 3
*Format*: docx. *Title*: Effect of ewe culling after reproductive failure on lifetime performance in favorable (ENV +) and unfavorable (ENV-) environments. Means, standard deviation (SD) and Spearman correlations (φ) are reported. Spearman correlations are only reported between culling scenarios within environment and within culling scenarios between environments. *Description*: A table showing results of a supplementary analysis to study the effect of ewe culling due to reproductive failure. In the simulation dataset used in the study (including 20 ewes replicates for 2760 individual types), In our simulation dataset, culling for reproductive failure was simulated by truncating ewe lifetime performances after the first null value for the number of lambs born.

## Data Availability

Supplementary Table is available in Supplementary Information 1. The data generated and/or analysed during the current study are available from the corresponding author on reasonable request. The code needed to run the model described in [14] is available at: 10.6084/m9.figshare.28840367.v3.
