# Supplementary material for: Breeding for sheep robustness: simulation of the consequences of ewe-lamb energy allocation trade-offs
Source: Genet Sel Evol. 2026 Apr 26;58:30. doi: 10.1186/s12711-026-01047-8 (PMC13262209; doi:10.1186/s12711-026-01047-8)
Supplement: Supplementary file 1 — Supplementary Material 1 Format: docx. Title: Influence of input‑parameter ranges on model outputs associated with their underlying bioenergetic processes. Description: A table summarizing the local effect of varying each input parameter within its defined range on a selected model output. For each parameter, the selected output is associated with a bioenergetic process that the parameter directly controls (even though parameters often control several processes). Parameters were varied independently between their lower and upper bounds while all others were kept at default values. Simulations were performed in the favorable environment. Details on parameters definitions and environmental scenarios are provided in Materials & Methods. [file 12711_2026_1047_MOESM1_ESM.docx]

**Additional file 1 Table S1**

Description: A table summarizing the local effect of varying each input parameter within its defined range on a selected model output. For each parameter, the selected output is associated with a bioenergetic process that the parameter directly controls (even though parameters often control several processes). Parameters were varied independently between their lower and upper bounds while all others were kept at default values. Simulations were performed in the favorable environment. Details on parameter definitions and environmental conditions are provided in Materials & Methods.

**Table S1**

**Title:** **Influence of input‑parameter ranges on model outputs associated with their underlying bioenergetic processes**

| **Process** | **Input parameter** | **Min** | **Max** | **Selected output** | **Unit** | **Input Min** | **Input**  **Max** |
| --- | --- | --- | --- | --- | --- | --- | --- |
| Acquisition | $b_{AcqStruct}$ | 0.23 | 0.28 | Lamb feed intake (dry matter) at weaning | g/d | 658 | 844 |
|  | ${PropRes}^{*}$ | 0.20 | 0.30 |  |  | 597 | 756 |
| Growth | ${MassStruct}^{*}$ | 6.00 | 7.00 | Lamb body mass at weaning (twins born from multiparous) | kg | 24.9 | 28.5 |
|  | ${AllocGrowth}^{*}$ | 0.50 | 0.70 |  |  | 26.4 | 31.0 |
|  | $k_{AllocGrowth_{U}}$ | 0.20 | 0.33 |  |  | 23.4 | 27.4 |
| Storage | ${AllocResProt}^{*}$ | 0.05 | 0.15 | Ewe BCS at first mating | pts | 2.6 | 3.4 |
| Sensitivity to body reserves variation | $b_{AcqPropRes}$ | 0.10 | 0.30 | Difference in ewe BCS between multiparous weaning 1 lamb vs. 3 lambs | pts | +0.69 | +0.31 |
|  | $b_{AllocPropRes}$ | 1.5 | 3.0 |  |  | +0.63 | +0.38 |
| Pregnancy | ${ovulrate}^{*}$ | 1.2 | 3.2 | NLB of multiparous ewes | n | 1.37 | 2.97 |
|  | $b_{AcqPreg}$ | 0.02 | 0.08 | Lamb mass at birth (twins born from multiparous) | kg | 4.12 | 4.78 |
|  | ${AllocPreg}^{*}$ | 0.40 | 0.70 |  |  | 3.62 | 5.27 |
|  | $k_{AllocPreg_{time}}$ | 2.5 | 4.0 |  |  | 4.44 | 3.05 |
|  | $pNL1_{Preg}$ | 0.35 | 0.75 |  |  | 5.47 | 3.34 |
|  | $pNL2_{Preg}$ | 0.80 | 1.00 |  |  | 4.34 | 5.00 |
| Lactation | $b_{AcqLact}$ | 0.50 | 1.30 | Lamb body mass at weaning (twins born from multiparous) | kg | 24.7 | 27.4 |
|  | $k1_{AcqLact}$ | 0.025 | 0.045 |  |  | 26.4 | 27.0 |
|  | $k2_{AcqLact}$ | 0.020 | 0.050 |  |  | 26.9 | 25.1 |
|  | ${AllocLact}^{*}$ | 0.50 | 1.00 |  |  | 23.0 | 26.7 |
|  | $k_{AllocLact_{time}}$ | 0.0025 | 0.0100 |  |  | 27.1 | 25.3 |
|  | $k_{AllocLact_{U}}$ | 0.30 | 3.00 | Lamb body mass at weaning (singleton born from primiparous) | kg | 31.0 | 31.7 |
|  | $pNL1_{Lact}$ | 0.45 | 0.75 | Lamb body mass at weaning (twins born from multiparous) | kg | 28.1 | 24.4 |
|  | $pNL2_{Lact}$ | 0.80 | 1.00 |  |  | 26.4 | 28.3 |
