# Supplementary material for: Breeding for sheep robustness: simulation of the consequences of ewe-lamb energy allocation trade-offs
Source: Genet Sel Evol. 2026 Apr 26;58:30. doi: 10.1186/s12711-026-01047-8 (PMC13262209; doi:10.1186/s12711-026-01047-8)
Supplement: Supplementary file 2 — Supplementary Material 2 Format: docx. Title: Table S2 Sensitivity indices of lifetime performance, neonate survival and ewe longevity. Description: A table reporting effect size estimates of varying the 22 input parameters on total number of lambs weaned (TNLW), neonate survival, and ewe longevity in the favorable (ENV +) and unfavorable (ENV-) environments. The 22 input parameters are defined in Table 1. Estimates of the sensitivity indices include linear effects (µ*) and non-linear and interaction effects (σ). The greater the values the more influence that parameter has on output traits. Estimations of effect size are based on 120 repetitions in each environment with the Morris's elementary effects screening method. [file 12711_2026_1047_MOESM2_ESM.docx]

**Additional file 2 Table S2**

Description: A table reporting effect size estimates of varying the 22 input parameters on total number of lambs weaned (TNLW), neonate survival, and ewe longevity in the favorable (ENV+) and unfavorable (ENV-) environments. The 22 input parameters are defined in Table 1. Estimates of the sensitivity indices include linear effects (µ*) and non-linear and interaction effects (σ). The greater the values the more influence that parameter has on output traits. Estimations of effect size are based on 120 repetitions in each environment with the Morris's elementary effects screening method.

**Table S2**

**Title: Sensitivity indices of lifetime performance, neonate survival and ewe longevity**

| **Output trait** | **ENV+** | | | **ENV-** | | |
| --- | --- | --- | --- | --- | --- | --- |
|  | **Input parameter** | **μ*** | **σ** | **Input parameter** | **μ*** | **σ** |
| TNLW (n lambs) | ${ovulrate}^{*}$ | 7.53 | 4.54 | ${PropRes}^{*}$ | 5.77 | 3.76 |
|  | ${PropRes}^{*}$ | 4.43 | 4.53 | $b_{AcqStruct}$ | 4.47 | 3.27 |
|  | ${AllocPreg}^{*}$ | 3.73 | 4.33 | ${ovulrate}^{*}$ | 3.86 | 3.99 |
|  | $k_{AllocPreg_{time}}$ | 3.31 | 3.99 | ${AllocResProt}^{*}$ | 3.21 | 3.06 |
|  | $b_{AcqPropRes}$ | 3.24 | 4.15 | $pNL1_{Preg}$ | 2.85 | 3.57 |
|  | $b_{AcqStruct}$ | 3.18 | 3.86 | ${AllocPreg}^{*}$ | 2.72 | 3.56 |
|  | ${AllocResProt}^{*}$ | 2.92 | 3.56 | $pNL1_{Lact}$ | 2.28 | 2.88 |
|  | ${AllocLact}^{*}$ | 2.70 | 3.43 | $b_{AcqPropRes}$ | 2.24 | 2.74 |
|  | ${AllocGrowth}^{*}$ | 2.62 | 3.33 | ${AllocLact}^{*}$ | 2.18 | 2.40 |
|  | $pNL1_{Preg}$ | 2.62 | 3.39 | $k_{AllocPreg_{time}}$ | 2.18 | 2.94 |
|  | $b_{AllocPropRes}$ | 2.59 | 3.30 | ${MassStruct}^{*}$ | 2.14 | 2.70 |
|  | ${MassStruct}^{*}$ | 2.56 | 3.15 | $b_{AcqLact}$ | 2.02 | 2.64 |
|  | $b_{AcqPreg}$ | 2.49 | 3.22 | $k1_{AcqLact}$ | 1.94 | 2.45 |
|  | $b_{AcqLact}$ | 2.41 | 3.09 | $k_{AllocLact_{time}}$ | 1.88 | 2.41 |
|  | $k_{AllocLact_{U}}$ | 2.33 | 3.04 | $b_{AllocPropRes}$ | 1.88 | 2.66 |
|  | $pNL1_{Lact}$ | 2.32 | 2.82 | $k_{AllocLact_{U}}$ | 1.85 | 2.46 |
|  | $pNL2_{Preg}$ | 2.26 | 2.98 | $k2_{AcqLact}$ | 1.82 | 2.38 |
|  | $k1_{AcqLact}$ | 2.20 | 2.85 | $pNL2_{Preg}$ | 1.81 | 2.39 |
|  | $pNL2_{Lact}$ | 2.11 | 2.76 | ${AllocGrowth}^{*}$ | 1.76 | 2.21 |
|  | $k2_{AcqLact}$ | 2.06 | 2.89 | $pNL2_{Lact}$ | 1.76 | 2.32 |
|  | $k_{AllocGrowth_{U}}$ | 2.04 | 2.78 | $b_{AcqPreg}$ | 1.73 | 2.40 |
|  | $k_{AllocLact_{time}}$ | 2.00 | 2.78 | $k_{AllocGrowth_{U}}$ | 1.64 | 2.10 |
| neonatal survival (%) | ${ovulrate}^{*}$ | 20.6 | 25.9 | ${ovulrate}^{*}$ | 27.4 | 21.9 |
|  | ${AllocPreg}^{*}$ | 17.4 | 21.9 | $pNL1_{Preg}$ | 23.5 | 16.9 |
|  | $k_{AllocPreg_{time}}$ | 14.9 | 20.6 | $b_{AcqStruct}$ | 21.0 | 18.3 |
|  | $pNL1_{Preg}$ | 13.8 | 15.3 | ${AllocPreg}^{*}$ | 18.1 | 18.2 |
|  | $b_{AcqPropRes}$ | 10.3 | 13.5 | $k_{AllocPreg_{time}}$ | 15.6 | 15.3 |
|  | $b_{AcqStruct}$ | 9.4 | 12.2 | ${AllocResProt}^{*}$ | 13.0 | 15.3 |
|  | ${MassStruct}^{*}$ | 7.6 | 9.7 | $b_{AcqPreg}$ | 9.5 | 11.0 |
|  | $b_{AcqPreg}$ | 6.4 | 8.6 | $b_{AcqPropRes}$ | 9.2 | 11.5 |
|  | ${AllocResProt}^{*}$ | 6.0 | 8.4 | ${PropRes}^{*}$ | 8.9 | 11.8 |
|  | ${PropRes}^{*}$ | 5.7 | 7.2 | ${MassStruct}^{*}$ | 8.7 | 9.9 |
|  | $b_{AllocPropRes}$ | 5.4 | 7.3 | $k_{AllocGrowth_{U}}$ | 7.8 | 9.7 |
|  | ${AllocGrowth}^{*}$ | 5.1 | 7.1 | $b_{AllocPropRes}$ | 7.8 | 9.7 |
|  | $pNL2_{Preg}$ | 5.1 | 6.7 | $pNL1_{Lact}$ | 7.0 | 9.4 |
|  | ${AllocLact}^{*}$ | 4.9 | 6.9 | $b_{AcqLact}$ | 6.9 | 9.1 |
|  | $pNL2_{Lact}$ | 4.9 | 6.3 | $pNL2_{Lact}$ | 6.3 | 8.5 |
|  | $k_{AllocGrowth_{U}}$ | 4.6 | 6.5 | $k_{AllocLact_{U}}$ | 6.2 | 8.6 |
|  | $k2_{AcqLact}$ | 4.5 | 6.0 | ${AllocGrowth}^{*}$ | 6.2 | 8.9 |
|  | $k_{AllocLact_{time}}$ | 4.4 | 5.7 | $k_{AllocLact_{time}}$ | 6.2 | 8.1 |
|  | $b_{AcqLact}$ | 4.2 | 5.8 | $k2_{AcqLact}$ | 6.0 | 7.6 |
|  | $k_{AllocLact_{U}}$ | 4.1 | 5.5 | $pNL2_{Preg}$ | 6.0 | 7.7 |
|  | $k1_{AcqLact}$ | 3.9 | 5.0 | $k1_{AcqLact}$ | 5.4 | 7.4 |
|  | $pNL1_{Lact}$ | 3.8 | 4.9 | ${AllocLact}^{*}$ | 5.4 | 7.2 |
| ewe longevity (n parities) | ${PropRes}^{*}$ | 2.61 | 2.18 | ${PropRes}^{*}$ | 4.10 | 1.79 |
|  | ${AllocPreg}^{*}$ | 1.92 | 2.09 | $b_{AcqStruct}$ | 2.05 | 1.79 |
|  | $k_{AllocPreg_{time}}$ | 1.82 | 2.09 | ${AllocResProt}^{*}$ | 1.64 | 1.68 |
|  | ${AllocResProt}^{*}$ | 1.75 | 1.79 | ${AllocLact}^{*}$ | 1.47 | 1.62 |
|  | ${ovulrate}^{*}$ | 1.58 | 2.06 | ${AllocPreg}^{*}$ | 1.44 | 1.73 |
|  | $b_{AcqPropRes}$ | 1.58 | 1.93 | ${MassStruct}^{*}$ | 1.38 | 1.68 |
|  | $b_{AcqStruct}$ | 1.53 | 1.83 | $pNL1_{Lact}$ | 1.36 | 1.61 |
|  | ${AllocLact}^{*}$ | 1.53 | 1.81 | $b_{AcqPropRes}$ | 1.35 | 1.55 |
|  | $b_{AllocPropRes}$ | 1.48 | 1.76 | $b_{AcqLact}$ | 1.35 | 1.76 |
|  | ${AllocGrowth}^{*}$ | 1.46 | 1.78 | ${ovulrate}^{*}$ | 1.33 | 1.64 |
|  | $b_{AcqPreg}$ | 1.41 | 1.74 | $k_{AllocPreg_{time}}$ | 1.32 | 1.71 |
|  | ${MassStruct}^{*}$ | 1.38 | 1.74 | $pNL1_{Preg}$ | 1.29 | 1.57 |
|  | $b_{AcqLact}$ | 1.34 | 1.57 | $k_{AllocLact_{time}}$ | 1.23 | 1.49 |
|  | $pNL2_{Preg}$ | 1.32 | 1.69 | $b_{AllocPropRes}$ | 1.22 | 1.62 |
|  | $pNL1_{Preg}$ | 1.30 | 1.55 | $k1_{AcqLact}$ | 1.21 | 1.58 |
|  | $pNL1_{Lact}$ | 1.29 | 1.47 | $k_{AllocLact_{U}}$ | 1.19 | 1.54 |
|  | $k_{AllocLact_{U}}$ | 1.22 | 1.53 | $k2_{AcqLact}$ | 1.19 | 1.49 |
|  | $pNL2_{Lact}$ | 1.21 | 1.52 | $b_{AcqPreg}$ | 1.18 | 1.52 |
|  | $k_{AllocGrowth_{U}}$ | 1.19 | 1.58 | $pNL2_{Preg}$ | 1.17 | 1.51 |
|  | $k1_{AcqLact}$ | 1.18 | 1.48 | ${AllocGrowth}^{*}$ | 1.17 | 1.46 |
|  | $k2_{AcqLact}$ | 1.16 | 1.46 | $k_{AllocGrowth_{U}}$ | 1.16 | 1.42 |
|  | $k_{AllocLact_{time}}$ | 1.12 | 1.41 | $pNL2_{Lact}$ | 1.12 | 1.44 |
