# Supplementary material for: Breeding for sheep robustness: simulation of the consequences of ewe-lamb energy allocation trade-offs
Source: Genet Sel Evol. 2026 Apr 26;58:30. doi: 10.1186/s12711-026-01047-8 (PMC13262209; doi:10.1186/s12711-026-01047-8)
Supplement: Supplementary file 3 — Supplementary Material 3 Format: docx. Title: Effect of ewe culling after reproductive failure on lifetime performance in favorable (ENV +) and unfavorable (ENV-) environments. Means, standard deviation (SD) and Spearman correlations (φ) are reported. Spearman correlations are only reported between culling scenarios within environment and within culling scenarios between environments. Description: A table showing results of a supplementary analysis to study the effect of ewe culling due to reproductive failure. In the simulation dataset used in the study (including 20 ewes replicates for 2760 individual types), In our simulation dataset, culling for reproductive failure was simulated by truncating ewe lifetime performances after the first null value for the number of lambs born. [file 12711_2026_1047_MOESM3_ESM.docx]

**Additional file 3 Table S3**

Description: A table showing results of a supplementary analysis to study the effect of ewe culling due to reproductive failure. In the simulation dataset used in the study (including 20 ewes replicates for 2760 individual types), In our simulation dataset, culling for reproductive failure was simulated by truncating ewe lifetime performances after the first null value for the number of lambs born.

**Table S3**

**Title:** **Effect of ewe culling after reproductive failure on lifetime performance in favorable (ENV+) and unfavorable (ENV-) environments. Means, standard deviation (SD) and Spearman correlations (φ) are reported. Spearman correlations are only reported between culling scenarios within environment and within culling scenarios between environments.**

| Case | Trait | ENV | Culling for reproductive failure | Mean | SD | φ | | | |
| --- | --- | --- | --- | --- | --- | --- | --- | --- | --- |
|  |  |  |  |  |  | NC+ | NC- | C+ | C- |
| NC+ | TNLW | ENV+ | No | 9.95 | 4.04 |  | - | - | - |
| NC- |  | ENV- |  | 5.75 | 3.55 | **0.54** |  | - | - |
| C+ |  | ENV+ | Yes | 7.15 | 3.96 | 0.94 | - |  | - |
| C- |  | ENV- |  | 3.26 | 2.95 | - | 0.93 | **0.48** |  |
| NC+ | TNLB | ENV+ | No | 12.16 | 4.68 |  | - | - | - |
| NC- |  | ENV- |  | 8.19 | 4.77 | **0.69** |  | - | - |
| C+ |  | ENV+ | Yes | 8.72 | 4.06 | 0.94 | - |  | - |
| C- |  | ENV- |  | 4.82 | 4.72 | - | 0.94 | **0.61** |  |
| NC+ | Number of parities | ENV+ | No | 6.23 | 1.63 |  | - | - | - |
| NC- |  | ENV- |  | 4.10 | 1.97 | **0.67** |  | - | - |
| C+ |  | ENV+ | Yes | 4.44 | 1.94 | 0.94 | - |  | - |
| C- |  | ENV- |  | 2.44 | 1.76 | - | 0.96 | **0.58** |  |

TNLW = Total Number of lambs Weaned, TNLB = number of lambs Born
